# Supplementary material for: An expanding manifold in transmodal regions characterizes adolescent reconfiguration of structural connectome organization
Source: eLife. 2021 Mar 31;10:e64694. doi: 10.7554/eLife.64694 (PMC8087442; doi:10.7554/eLife.64694)
Supplement: Supplementary file 2. [file elife-64694-supp2.pdf]

Neuroscience in Psychiatry Network (NSPN) Consortium author list

Principal investigators:
Edward Bullmore (CI from 01/01/2017)[1,2,3]; Raymond Dolan[4,5]; Ian Goodyer (CI until 01/01/2017)[1]; Peter Fonagy[6]; Peter Jones[1]

NSPN (funded) staff:
Michael Moutoussis[4,5]; Tobias Hauser[4,5]; Sharon Neufeld[1]; Rafael Romero-Garcia[1,2]; Michelle St Clair[1]; Petra Vértes[1,2]; Kirstie Whitaker[1,2]; Becky Inkster[1]; Gita Prabhu[4,5]; Cinly Ooi[1]; Umar Toseeb[1]; Barry Widmer[1]; Junaid Bhatti[1]; Laura Villis[1]; Ayesha Alrumaithi[1]; Sarah Birt[1]; Aislinn Bowler[5]; Kalia Cleridou[5]; Hina Dadabhoy[5]; Emma Davies[1]; Ashlyn Firkins[1]; Sian Granville[5]; Elizabeth Harding[5]; Alexandra Hopkins[4,5]; Daniel Isaacs[5]; Janchai King[5]; Danae Kokorikou[5,6]; Christina Maurice[1]; Cleo McIntosh[1]; Jessica Memarzia[1]; Harriet Mills[5]; Ciara O'Donnell[1]; Sara Pantaleone[5]; Jenny Scott[1]; Beatrice Kiddle[1]; Ela Polek[1]

Affiliated scientists:
Pasco Fearon[6]; John Suckling[1]; Anne-Laura van Harmelen[1]; Rogier Kievit[4,7]; Sam Chamberlain[1]

[1]Department of Psychiatry, University of Cambridge, United Kingdom
[2]Behavioural and Clinical Neuroscience Institute, University of Cambridge, United Kingdom
[3]ImmunoPsychiatry, GlaxoSmithKline Research and Development, United Kingdom
[4]Max Planck University College London Centre for Computational Psychiatry and Ageing Research, University College London, UK
[5]Wellcome Centre for Human Neuroimaging, University College London, United Kingdom
[6]Research Department of Clinical, Educational and Health Psychology, University College London, United Kingdom
[7]Medical Research Council Cognition and Brain Sciences Unit, University of Cambridge, United Kingdom
